# Neuromesodermal progenitors are a conserved source of spinal cord with divergent growth dynamics

**DOI:** 10.1242/dev.166728

**Published:** 2018-11-09

**Authors:** Andrea Attardi, Timothy Fulton, Maria Florescu, Gopi Shah, Leila Muresan, Martin O. Lenz, Courtney Lancaster, Jan Huisken, Alexander van Oudenaarden, Benjamin Steventon

**Affiliations:** 1 Department of Genetics, University of Cambridge, Cambridge CB2 3EH, UK; 2 Max Planck Institute of Molecular Cell Biology and Genetics, Dresden 01307, Germany; 3 STEBICEF Department, Università degli Studi di Palermo, Palermo 90133, Italy; 4 Hubrecht Institute, Utrecht 3584 CT, The Netherlands; 5 European Molecular Biology Laboratory, Carrer del Dr. Aiguader, 88, 08003 Barcelona, Spain; 6 Cambridge Advanced Imaging Centre, Cambridge CB2 3EH, UK; 7 Morgridge Institute for Research, Madison, WI 53715, USA

**Keywords:** Zebrafish, Gastrulation, Tailbud, Axial elongation

## Abstract

During gastrulation, embryonic cells become specified into distinct germ layers. In mouse, this continues throughout somitogenesis from a population of bipotent stem cells called neuromesodermal progenitors (NMps). However, the degree of self-renewal associated with NMps in the fast-developing zebrafish embryo is unclear. Using a genetic clone-tracing method, we labelled early embryonic progenitors and found a strong clonal similarity between spinal cord and mesoderm tissues. We followed individual cell lineages using light-sheet imaging, revealing a common neuromesodermal lineage contribution to a subset of spinal cord tissue across the anterior-posterior body axis. An initial population subdivides at mid-gastrula stages and is directly allocated to neural and mesodermal compartments during gastrulation. A second population in the tailbud undergoes delayed allocation to contribute to the neural and mesodermal compartment only at late somitogenesis. Cell tracking and retrospective cell fate assignment at late somitogenesis stages reveal these cells to be a collection of mono-fated progenitors. Our results suggest that NMps are a conserved population of bipotential progenitors, the lineage of which varies in a species-specific manner due to vastly different rates of differentiation and growth.

## Abstract (precis)

**Highlighted Article:** A complete lineage analysis of neuromesodermal progenitors in zebrafish through a combination of genetic clonal labelling, forward fate mapping and *in toto* imaging.

## INTRODUCTION

In amniotes, a bipotent population of posterior progenitors continually allocate cells to the posterior pre-somitic mesoderm (PSM) and spinal cord (Tzouanacou et al., 2009; Brown and Storey, 2000; Selleck and Stern, 1991). Although no specific molecular marker has been identified for this population, they have been shown to express a combination of both the early neural and mesodermal markers Sox2 and brachyury (Wymeersch et al., 2016), and have been named ‘neuromesodermal progenitors’ (NMps; Henrique et al., 2015). The observation of a NMp population is important as it suggests that germ layer specification continues throughout somitogenesis stages and is not restricted to primary gastrulation. Understanding when NMps allocate cells to spinal cord and paraxial mesoderm is an essential first step in exploring the underlying molecular processes that determine the timing of germ layer allocation during vertebrate embryonic development.

The continuous allocation of cells from an NMp pool fits well with the cellular basis of axial elongation in mouse embryos, in which primary gastrulation generates only head structures and the rest of the body axis is generated by posterior growth (Steventon and Martinez Arias, 2017). However, externally developing embryos such as the zebrafish elongate their body axis in the absence of posterior volumetric growth, with elongation being a consequence of convergence and extension of mesodermal progenitors, and volumetric growth of tissue within the already segmented region of the body axis (Steventon et al., 2016). Despite these differences, genetic studies have revealed a marked conservation in the signals and gene regulatory networks that act to drive posterior body elongation across bilaterians (Martin and Kimelman, 2009). Furthermore, experiments in the zebrafish embryo have confirmed the presence of Sox2/Tbxta-positive cells in the tailbud, and transplantation of single cells into the marginal zone can be directed to either neural or mesodermal cell fates, depending on the level of canonical Wnt signalling (Martin and Kimelman, 2012). An additional progenitor pool exists within the tailbud that generates cells of the notochord and floorplate in a Wnt- and Notch-dependent manner (Row et al., 2016). Although these studies demonstrate that a neuromesodermal competent population exists within the zebrafish tailbud, lineage analysis using intracellular injection of high molecular weight fluorescent dextran (Kanki and Ho, 1997) argues against a stem cell-like population that is homologous to the mouse NMp pool (Tzouanacou et al., 2009). These seemingly conflicting results can be resolved by a complete lineage analysis that determines the timing of neural and mesodermal lineage restriction in zebrafish, and answer in a clear manner whether these progenitors arise from a stem cell pool as they do in mouse embryos.

We define ‘neuromesodermal progenitors/NMps’ to be a population of cells that are competent to produce either spinal cord or paraxial mesoderm fates, and that have developed beyond stages at which embryonic cells display pluripotency. Although it is clear from cell transplant studies in the zebrafish that a population of bipotent progenitors exist (Martin and Kimelman, 2012), the degree to which individual cells will divide and give rise to both cell fates is unknown. One possibility is that a neuromesodermal competent state is a conserved feature of vertebrate embryonic development, but how potential is realized is dependent on the degree of posterior growth associated with the species in question; an interesting hypothesis that suggests a dissociation between those molecular processes driving multipotency and those that lead to a clonal expansion of progenitor populations. Zebrafish offer an extreme system with which to explore this hypothesis owing to their rapid development and the minimal volumetric growth that is associated with their body axis elongation (Steventon et al., 2016). We therefore distinguish between the term ‘bipotential’ (reflecting a state of competence) from ‘mono-fated’ or ‘bi-fated’ cells (reflecting a retrospective assignment of fates during normal development by lineage tracing).

Here, we have used a genetic clone-tracing method to address whether zebrafish NMps are a conserved source of spinal cord tissue, and the degree to which they populate neural and mesodermal structures during normal development. We find a closer clonal relationship between spinal cord and muscle when compared with spinal cord and anterior neural regions, which can be explained by a model of NM lineage decision at the basis of spinal cord generation in zebrafish. Tracing this lineage restriction with the combined use of photolabelling and the single cell tracking of lineages from an *in toto* light-sheet imaging dataset demonstrate that this restriction occurs during an early and direct segregation event with little or no amplification of the cellular pool. We observe a second population of NMps that remains resident in the tailbud and contributes to the caudal-most region of the tail, which matches a previously described tailbud NMp population (Martin and Kimelman, 2012). Taken together with recent studies, this suggests that an NMp population is a conserved source of spinal cord and paraxial mesoderm, but with large differences in their potential for self-renewal *in vivo*.

## RESULTS

### Spinal cord scars are more closely associated to mesodermal than to anterior neural derivatives

**Fig. 1.**
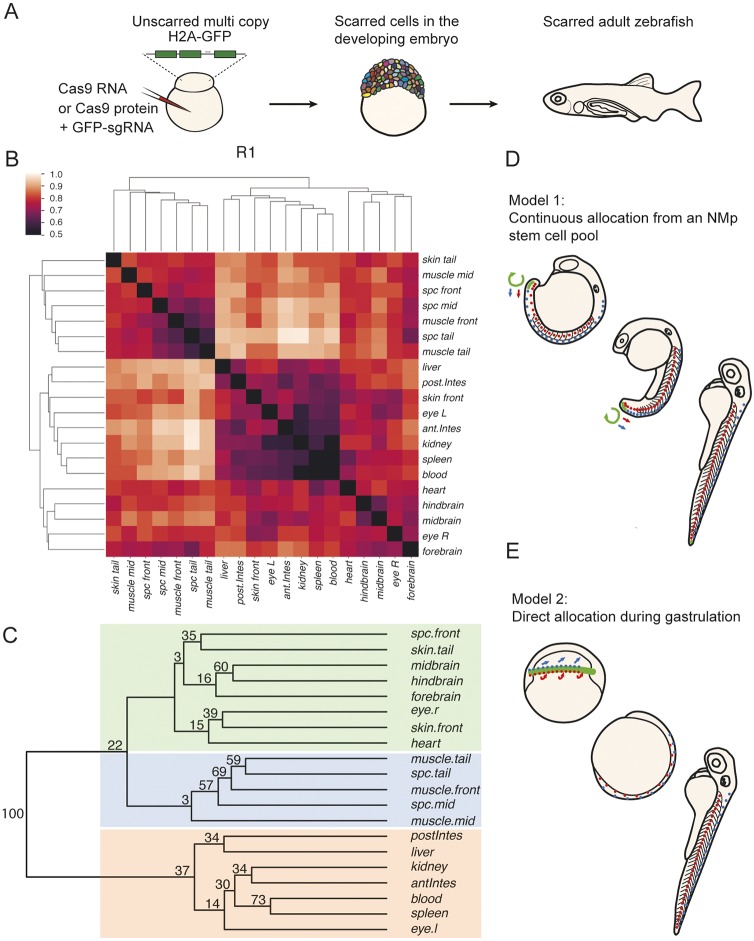
**Spinal cord clones are closely associated to mesodermal, but not anterior, neural derivatives.** (A) Experimental workflow for Scartrace lineage tracing of the zebrafish larvae. (B) Clustering of weighted distances (see Materials and Methods) of scars in dissected body structures of fish R1. The smaller the distance the closer the association between body structures. (C) Bootstrapped tree of weighted distances of scars in dissected body structures of fish R1. The tree was bootstrapped 100 times and the final proportion of clades (clade support values) are shown at each node of a clade. Coloured boxes highlight three groups: the ectodermal group (green), the mesodermal/endodermal group (orange), and the mixed ectodermal and mesodermal group (blue) containing muscle and spinal cord. There is close association of spinal cord and mesodermal cells across the anterior to posterior axis, that is distinct from brain regions. spc, spinal cord; antIntes, anterior intestine; postIntes, posterior intestine; l, left; r, right. (D) Model 1. The continuous allocation model is based on lineage analysis in mouse embryos and proposes that a group of self-renewing neuromesodermal stem cells continually generates spinal cord and paraxial mesoderm throughout axial elongation. (E) Model 2. The direct allocation model proposes that an early segregation of neural and mesodermal lineages occurs during gastrulation, the derivatives of which then expand rapidly by convergence and extension.

To determine whether there are shared progenitors between the spinal cord and mesodermal derivatives, such as muscle, at the whole-organism level, we used ScarTrace, a CRISPR/Cas9-based genetic clone-tracing method that labels clones uniquely in the developing embryo and allows for the reconstruction of clonal relationships in a retrospective manner (Alemany et al., 2018; Junker et al., 2017preprint). When Cas9 is injected together with a single-guide RNA (sgRNA) targeting a tandem array of histone-GFP transgenes in a zygote, a series of insertions and deletions of different lengths at different positions (scars) is introduced after double-stranded DNA breaks. These scars are inherited by all daughters of each labelled progenitor cell. If progenitors are shared between the spinal cord lineage and paraxial mesoderm tissues in zebrafish, it would be expected that descendants of the paraxial mesoderm tissues would have similar scars to those within the spinal cord. Alternatively, if spinal cord were generated from an ectodermal territory that is segregated from the mesoderm prior to anterior/posterior neuronal lineage segregation, as predicted by the activation/transformation model (Kiecker and Niehrs, 2001; Nieuwkoop and Nigtevecht, 1954), it would be expected that it would share a common set of scars with brain regions. To distinguish between these two possibilities, we injected either Cas9 RNA (fish R1 to R3) or Cas9 protein (fish P1 to P3) together with a sgRNA against GFP at the one-cell stage into embryos transgenic for eight copies of H2A-GFP (Fig. 1A). Cas9 RNA injection has been shown to generate scars up until 10 hpf (hours post fertilization) and therefore would label any neural and mesodermal progenitors throughout gastrulation, whereas Cas9 protein scarring ends at around 3 hpf (Alemany et al., 2018). We isolated and sequenced scars of a total of 140 regions of six adult fish (∼1 year post fertilization). We then computed the distance between organs using a weighted distance measure of binarized rare scars (see Materials and Methods), and displayed them as heat map and bootstrapped trees (Fig. 1B,C; Fig. S1). For Cas9 RNA-injected fish, we observe a smaller distance between spinal cord and muscle tissues at middle and tail regions of the body axis than to head regions (Fig. 1B; Fig. S1). This results in brain, anterior and skin grouping consistently together as a distinct group from the more posterior spinal cord and muscle populations (green and blue boxes, respectively in Fig. 1C and Fig. S1). Intestine, liver, kidney, blood and spleen all group together in a more distant third grouping (red box; Fig. 1C). For Cas9 protein-injected fish we observe a weaker grouping, with only a close association of spinal cord tissue across the anterior to posterior axis to muscle tissue at only P1 (Fig. S1). This is consistent with the early scarring of cells up until 3 hpf (Alemany et al., 2018).

This observation is compatible with two models for the segregation of spinal cord and paraxial mesoderm fates during zebrafish embryogenesis. The first model, continuous allocation, follows the interpretation of retrospective lineage analysis in the mouse (Fig. 1D) and assumes that both spinal cord and paraxial mesoderm cells are continually produced from a posteriorly localized neuromesodermal stem cell pool. The alternative is the early segregation model, according to which neural cell lineages diverge during early gastrulation stages, concomitant with the early specification of mesoderm tissue (Fig. 1E). To determine the relative contribution of these two segregation models of zebrafish NMps, we turned to imaging-based lineage-tracing methods.

### Tissue-level segregation of spinal cord and mesoderm populations occurs by 50% epiboly

**Fig. 2.**
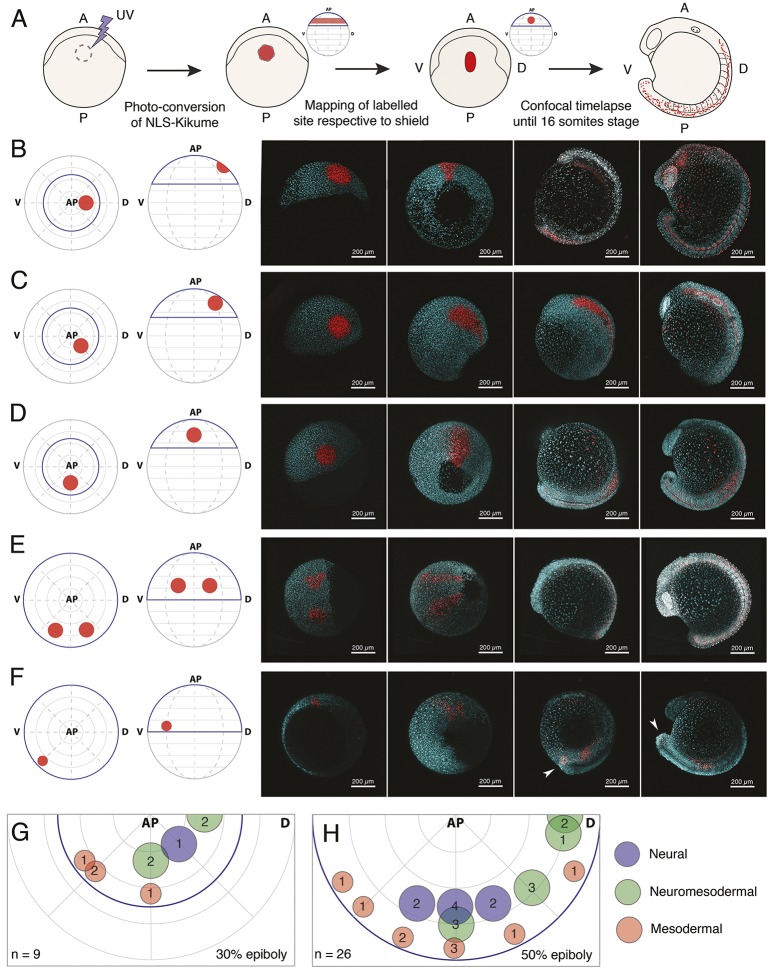
**Tissue-level segregation of spinal cord and mesoderm populations occurs by 50% epiboly.** (A) Description of large-scale fate-mapping experiments. NLS-Kik-injected embryos are mounted for confocal microscopy at 30 or 50% epiboly, and a circular region of the blastoderm is photoconverted. Time-lapse imaging is carried out until the 16-somite stage. Position of the label is retrospectively assigned based on its location on the anterior-posterior axis (determined at the moment of labelling) and the position of the prospective embryonic shield (appearing right after 50% epiboly), which marks the dorsal side of the embryo. (B-F) Embryos were photolabelled in the territories shown in the animal and lateral view diagrams (left-most image) and cells were followed until the 16-somite stage (right-most image panels) by time-lapse microscopy. Consecutive time points are displayed in the right-hand four panels for each example label. Labels were placed in the prospective anterior neural (B), marginal zone (C) and prospective spinal cord (D) territories at 30% epiboly. At 50% epiboly, the boundaries of the prospective spinal cord region (E) and a smaller, more ventro-marginal, mesodermal region (F) were mapped. Arrowheads in F indicate that a subset of labelled cells specifically migrates and contributes to the tailbud mesenchyme. Native NLS-KikGR is shown in cyan, photoconverted NLS-Kik in red. (G,H) Plots showing the location and diameter of labels in relation to the number of embryos imaged per label at 30% (G) and 50% (H) epiboly. Labels are colour-coded according to the ratio of cells allocated to neural tissues or paraxial mesoderm. Blue indicates that over 90% of labelled cells contribute to neural tissues; red indicates that over 90% of labelled cells contribute to paraxial mesoderm; green indicates that labelled cells contribute both to neural and paraxial mesoderm. *n* indicates total number of embryos fate mapped. AP, animal pole; V, prospective ventral side; D, prospective dorsal side (shield). Dorsal and ventral only indicate 3D orientation of the embryo and not future dorsoventral position of cells.

**Fig. 3.**
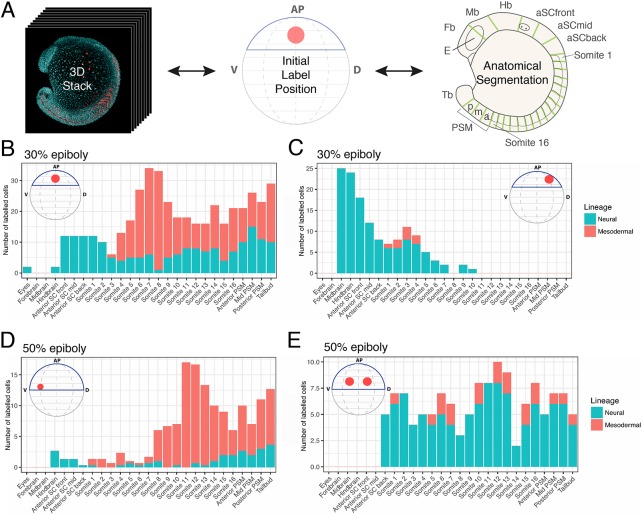
**Axial dispersion and neuro-mesodermal contribution of labelled cells.** (A) 3D confocal stacks of photolabelled embryos were analysed to relate the initial label position with the contribution of cells along the anterior-posterior axis. (B-E) The contributions of labelled populations from individual examples are plotted against the anterior-posterior axis with the number of cells in each tissue compartment shown in red for the somitic mesoderm or blue for the neural tube. There is a significant degree of overlap between spinal cord- and mesoderm-fated cells within the marginal zone at both 30% (B,C) and 50% (D,E) epiboly.

To obtain an estimate of when spinal cord and paraxial mesoderm spatially segregate during zebrafish gastrulation, we performed a series of fate-mapping experiments using photolabelling. Embryos were injected at the one-cell stage with mRNA encoding for the photoconvertible protein, Kikume, targeted to the nucleus (nls-Kikume). Upon exposure to UV light at 30% or 50% epiboly, circular patches of blastodermal cells close to the marginal zone were labelled, enabling the direct visualization of mesoderm and ectoderm segregation, migration and tissue contribution by confocal microscopy (Fig. 2A; Movie 1). Although labels in the prospective anterior-dorsal region of the 30% epiboly embryo contributed to large regions of the brain and notochord (Fig. 2B), these labels contributed little to the spinal cord and paraxial mesoderm territories, supporting the distinct lineage for brain regions found by Scartrace (Fig. 1A,B). The co-labelling of anterior neural- and notochord-fated cells reflects the fact that the labelled ectodermal domain partially overlaps with the prospective shield region at 30% epiboly. A region of cells adjacent to the anterior neural domain contributes to midbrain, hindbrain and the most anterior part of the spinal cord (Figs 2C and 3C), again consistent with the grouping of anterior spinal cord scars with the brain regions (Fig. 1C; green box). Labels along the medial regions of the marginal zone generate significant contributions to not only the spinal cord, but also paraxial mesoderm compartments (Fig. 2D, Movie 1). Labels spanning the margins of this prospective spinal cord domain performed at 50% epiboly result in significantly fewer mesodermal contributions, in line with the continued invagination of mesodermal progenitors at these stages (Figs 2E and 3B,E). However, not even small (36 cells) labels could mark solely mesoderm-fated cells, suggesting that a degree of mixing between ectodermal and mesodermal lineages persists until 50% epiboly (Figs 2F and 3D). A summary of all labels in terms of their contribution to neural tissues only (blue spots), mesodermal only (red spots) or both neural and mesoderm (green spots) can be observed after mapping the position of each label with respect to the embryonic shield and animal pole for labels at 30% (Fig. 2G) and 50% (Fig. 2H) epiboly.

Following the 50% spinal cord/mesoderm-fated populations by time-lapse microscopy reveals a rapid convergence and extension of spinal cord progenitors that leads to a widespread contribution across a large proportion of the anterior-posterior axis (Movies 2 and 3). Contributions of each label were counted for somite and corresponding neural segments at the 16-somite stage (Fig. 3A), and displayed as histograms with the most anterior segment to the left of each plot (Fig. 3B-E). This shows how cells around the centre of the dorsal-to-ventral axis will contribute to neural tissue from the base of the hindbrain to the tailbud at the 16-somite stage (Fig. 3E). Cells that remain ectodermal upon invagination of the mesoderm become displaced posteriorly by the continued convergence and extension of cells in the animal pole (Movie 4). Thus, it appears that a large proportion of the spinal cord is allocated during gastrulation stages, and that this arises from a domain close to or overlapping with paraxial mesoderm-fated cells. However, in absence of single cell resolution, it is not possible to conclude whether these cells are a mixed population of mono-fated progenitors, or arise from a bi-fated neuromesodermal population.

### A mixed population of mono-fated and bi-fated neuromesodermal cells segregates rapidly during mid to late gastrulation

**Fig. 4.**
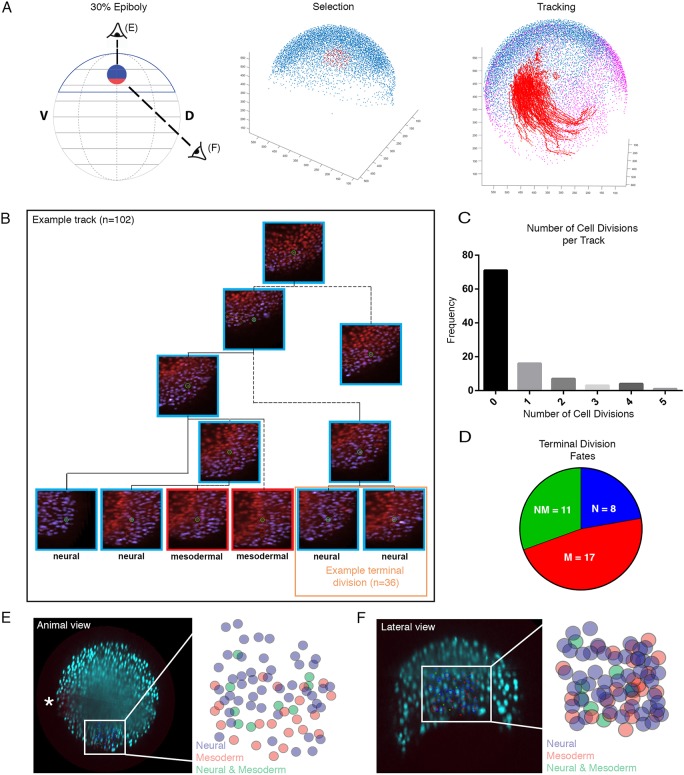
**Bipotential neuromesodermal cells segregate rapidly during mid to late gastrulation.** (A) *In silico* tracking of cells within the multi-view reconstruction was performed to follow lineage segregation from 30% epiboly. A custom MATLAB-based tool was developed to allow for the extraction of tracking data. Tracks were selected separately (*n*=100) in marginal and animal subgroups and visualized on top of 75% epiboly stage nuclei as a reference (purple). Cells were selected and tracked forwards in time, then scored based on expression or absence of mezzo:eGFP marking mesoderm. (B) An example track demonstrating a number of cell divisions and the two terminal divisions, one scored as both neural and one as a bi-fated NMp. In total 102 tracks were validated, with 36 terminal divisions scored. (C) Frequencies of cell divisions per track. (D) Of the 36 terminal divisions, 11 demonstrated contributions to both the neural and mesodermal compartment, with a remaining eight contributing to only neural and 17 to only the mesoderm. (E-F) From the animal and lateral views, the bi-fated cells are clearly intermixed with mono-fated cells. The fated cells are shown overlaid on the animal view image and then expanded in the schematic. Asterisk in E indicates the first mezzo:eGFP-positive cells and therefore the dorsal-most aspect of the marginal zone.

To assess whether single cells contribute to both spinal cord and mesoderm, we made use of an existing light-sheet dataset in which the onset of mesoderm specification can be observed with the use of a live reporter for *mezzo* (Shah et al., 2017preprint). In this dataset, germ layer segregation can be assessed live by detecting the increase in mezzo:eGFP fluorescence levels in the nuclei of mesendodermally specified cells (Fig. 4A). In the dataset used for tracking, a red channel is obtained to make mesodermal cells by taking all cells that are mezzo:eGFP positive and subtracting Sox17^+^ cells that are fated towards endoderm. Similarly, a blue channel is created for ectodermal cells that results from cells expressing the ubiquitous h2b-rfp and subtracting from all those that are mezzo:eGFP^+^ (Shah et al., 2017preprint). After segmentation and automated tracking of all nuclei within the gastrulating embryo, a custom MATLAB script was used to isolate tracks of cells in a user-defined region of the embryo at a chosen timepoint, thus allowing us to perform labelling experiments *in silico*. Automated cell tracking performed using TGMM (Amat et al., 2014) was validated by manually inspecting each track using the Fiji plug-in Mamut, by following each track and its associated cells through both time and z-slices (Wolff et al., 2018). A ‘track’ is defined as the sequence of connected x,y,z,t values associated with a cell and all of its progeny.

To characterize the germ layer allocation of individual cells within the medial marginal zone at 30% epiboly, we focused on a region comparable with the clone of cells fated towards both spinal cord and paraxial mesoderm (Fig. 2D). We hypothesized that the more-animal part of this region could consist of bipotent progenitors. In fact, separation of the tracks of marginal cells from the rest of cells present in the medial region shows that overall track lengths of marginal cells are shorter than those of more-animal cells, all ending before shield stage. Shorter track lengths of these cells is a consequence of their rapid mesodermal segregation though marginal involution (Movie 5). We therefore focused on the more-animal cells of the clone, that continued up to the end of epiboly (Fig. 4A).

Out of a total of 102 manually validated tracks from this region, 75% were assessed as being accurate without a requirement for manual correction. Twenty-three percent required manual correction, and the remaining tracks were discarded from the analysis due to gross inaccuracies in tracking. Cells that retained ectodermal lineage at track termination were re-selected; their final positions within the spinal cord could be observed at later stages (Fig. S2) and are shown by a blue box in Fig. 4B. Those that started expressing mezzo:eGFP (but not Sox17) were determined as being of mesoderm fate (Movie 5) and are indicated by a red box (Fig. 4B).

Over the duration of the movie analysis (from 30% epiboly to shield stage), 70% of tracks did not undergo a division and were thus directly determined as either being mono-fated spinal cord or paraxial mesoderm progenitors. The remaining tracks divided between one and five times over the duration of the analysis (Fig. 4C). To assign their fates, we focused on the terminal division progeny (Fig. 4B, orange box). Out of a total of 36 terminal divisions, 11 gave rise to both neural and mesodermal derivatives, with the remaining eight mono-fated as neural and 17 as mesodermal (Fig. 4D). Based on this, we conclude that a small population of bi-fated neuromesodermal progenitors exist close to the marginal zone of the zebrafish embryo, but rapidly segregate between the 70-90% epiboly stage.

To assess the degree of mixing between either mono-fated or bi-fated cells, we colour-coded the tracks according to their retrospectively defined fates and plotted them at 30% epiboly according to either an animal view (Fig. 4A,E) or lateral view (Fig. 4A,F). Enlargement of these fate maps reveal a significant degree of mixing between neural-, mesoderm- and neuromesodermal-fated cells along both the lateral-medial (Fig. 4E) and animal-vegetal (Fig. 4F) axes.

### Tailbud neuromesodermal populations are restricted to the posterior-most aspect of the body axis

**Fig. 5.**
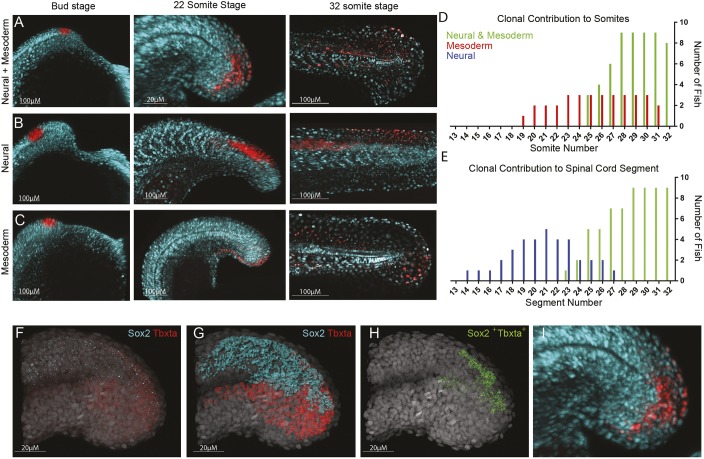
**Tailbud neuromesodermal populations are restricted to the posterior-most aspect of the body axis.** (A-C) Regions of the tailbud were photolabelled at bud stage and followed until the completion of somitogenesis. Populations on the dorsolateral wall of the tailbud gave rise to both spinal cord and mesoderm derivatives (A), those more anterior generated only spinal cord (B) and those more ventral gave rise to only mesoderm (C). All labels had an additional contribution to non-neural ectoderm. (D) The contribution of labelled populations to the anterior-posterior axis are plotted separately for mesodermal (D) and neural (E) fates. In red are labels with mesodermal contribution only (*n*=7), in blue are spinal cord-specific populations (*n*=5) and in green are those that gave rise to both germ layers (*n*=9). (F-I) Hybridization chain reaction (HCR) for *sox2* and *tbxta* was used to locate double-positive cells within 3D confocal datasets. (F) Original dataset with *sox2* in cyan and *tbxta* in red; DAPI is in grey. (G) Surface segmentation of the HCR stain was performed to mask *sox2* expression within the *tbxta* channel. (H) Masking reveals only those cells that are co-expressing both genes. (I) Magnified image of that shown in A to compare photolabel with co-expressing cells.

Upon completion of gastrulation, the tailbud forms via a continued convergence and extension of cells from the anterior ectoderm and a concomitant subduction of posterior cells to form the paraxial mesoderm (Kanki and Ho, 1997). The posterior-most subset of the labelled gastrula-stage NMp population becomes located within the dorsal tailbud upon the completion of primary gastrulation (Movie 3), suggesting that there may be a spatial continuity with the Sox2/Tbxta-positive population in this region (Martin and Kimelman, 2012). We used photo-labelling to assess the contribution of this population. Using nls-Kik-injected embryos, multiple regions of the tailbud were photolabelled at the 6-somite stage and then re-imaged at the end of somitogenesis; an intermediate image was taken at the 22-somite stage. A progenitor region that gives rise to both neural and mesodermal tissue was identified dorsal to the posterior end of the notochord (Fig. 5A). In tracking these cells, it was shown that between the 6- and 22-somite stages, the progenitor cells track the dorsoposterior end of the notochord and show no contribution to the extending axis. The population was also observed to undergo a dorsal-to-ventral rotational movement along the posterior wall, aligning with the caudal neural hinge. The NM-fated populations (*n*=9) were observed contributing to only the final seven somites (25th to 32nd) and nine neural segments (23rd to 32nd).

Directly adjacent anteriorly to the NM-fated region, a progenitor pool with only neural fate was identified. This population contributed to earlier forming spinal cord and no mesodermal tissue. Labelled neural populations were shown to contribute to neural segments between the 14th and 27th somites (*n*=7). No mesoderm was labelled in these labelled populations. Directly posteriorly adjacent to the neural mesoderm progenitors, a third population of progenitors was identified that contributed to only mesodermal tissues, in particular the earlier forming somites between the 19th and 31st (*n*=3).

To check whether our labels are covering the Sox2^+^Tbxta^+^ (previously ntl)^+^ domain previously described (Martin and Kimelman, 2012), we compared the labels with HCR stains for these genes (Choi et al., 2014; Fig. 5F). By segmenting each expression domain, the region of overlap can be visible (Fig. 5G; Movie 5). The *tbxta* expression domain was used to mask all *sox2* expression outside of this region of interest, thereby allowing only regions with co-expression to be observed in 3D (Fig. 5H; Movie 6). This region overlaps with the example NM-labelled population shown as an enlarged version in Fig. 5A,I.

**Fig. 6.**
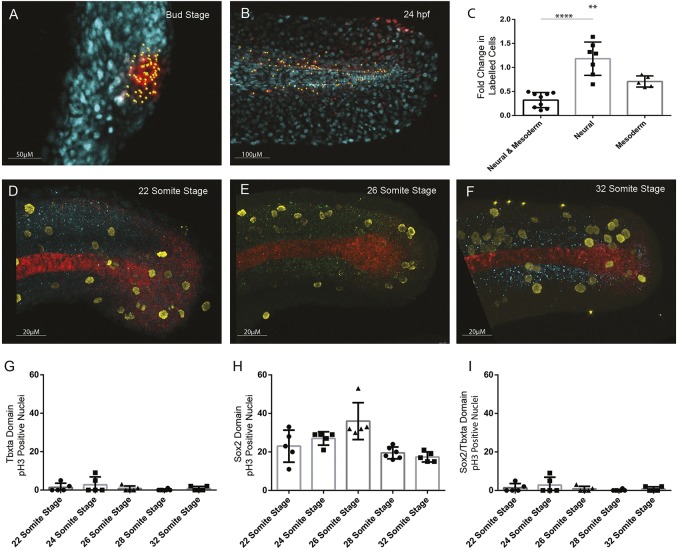
**Quantification of cell division in tailbud NMps.** Quantification of increase in number of cells photolabelled using nls-kikume from (A) bud stage through to (B) 24 hpf. (C) Fold-change increase in labelled clone number changes depending on the labelled progenitor type, with regions contributing only to neural tissue undergoing most clonal expansion and bipotent progenitors undergoing the least clonal expansion. (D-F) Replicating cells stained using phospho-histone H3 (pH3) as a marker of mitotic cells (yellow) with bipotent NMps identified through co-expression of Sox2 (blue) and Ntl (red) at the (D) 22-somite stage, (E) 26-somite stage and (F) 32-somite stage. (G-I) The frequency of Ph3-positive nuclei in the (G) Ntl-positive expression domain, including notochord, (H) Sox2-positive expression domain, and (I) Ntl and Sox2-positive NMp domain. Two-tailed Student's *t*-test. **P<0.01, ****P<0.0001.

By labelling the early progenitor populations and identifying their final fates at the end of somitogenesis (Fig. 5A-E), the number of cellular divisions was calculated (Fig. 6A-C). Neural- and mesoderm-specific progenitor populations were shown to both have a significantly (*P*<0.001, *n*=7) higher rate of proliferation than the neuromesodermal populations, which were shown to replicate infrequently. The mono-fated populations (MPs and NPs) underwent a much higher frequency of cell divisions, with NPs doubling in cell number between bud stage and the end of somitogenesis. These differences are matched by phopho-histone H3 antibody staining to quantify nuclei undergoing mitosis (Fig. 6D-F) and comparing division rates between *tbxta*-positive notochord (Fig. 6G), *sox2*-positive neural tissue (Fig. 6H) and co-expressing cells (Fig. 6I).

### Zebrafish tailbud neuromesodermal progenitors are a population of mono-fated cells

**Fig. 7.**
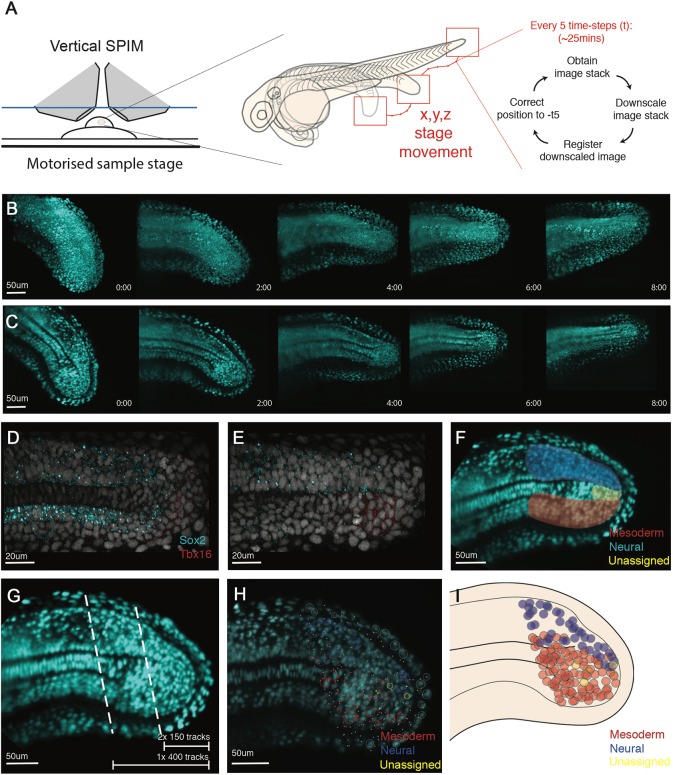
**Single cell tracking of tailbud progenitors during late somitogenesis demonstrates an absence of bi-fated cells and cellular mixing within the tailbud.** (A) Experimental design to allow long-term light-sheet imaging of the growing zebrafish tailbud. Embryos at the 21-somite stage were mounted as described by Hirsinger and Steventon (2017). *Z*-stacks were captured every 2.5 min with image-based registration on downsampled images conducted every five frames. The shift of this registration is then fed back into the stage position in all three directions to re-centre the tailbud within the image. (B,C) Individual frames are shown every 2 h across the 8 h movie shown as both maximum projections (B) and as a medial slice (C). (D-G) Fates of cells were assigned by the termination point of the track by scoring-based position within the anatomy of the tailbud and the associated expression of neural markers (Sox2) and mesdoermal markers (Tbx16 and Tbxta). Medial slices of hybridization chain reaction are shown in D and E, and used to zone the tailbud in F. Automatic tracks were selected using a custom MATLAB script by selecting all cells posteriorly to the lines shown in G. Two movies were subsetted using the most-posterior lines generating two movies of 150 tracks each. A third movie was subset using the more-anterior line, generating 400 tracks. (H,I) Final fates were assigned and have been overlaid over the starting timepoint (H) with open circles representing cells close to the viewed plane and dots representing cells on a different *z*-section. These different *z*-planes have been collapsed into a single 2D image (I) and demonstrate an absence of a mixed population of unfated cells. The mixing around the notochord is an artifact of collapsing the *z*-axis into a 2D image. No cells were observed dividing with progeny entering both lineages.

Following zebrafish tailbud at single-cell resolution is a difficult task due to the rapid displacement of the tailbud, which continually moves the region of interest outside the field of view. To compensate for this, we adapted a vertical light-sheet set-up to allow for a continuous correction of the microscope stage position such that the tailbud is continually kept between the excitation and detection objectives (Fig. 7A). As neuromesodermal contributions are restricted to the last seven to nine somite segments (Fig. 5), we focused on generating time-lapse data from the 21 somites through to the completion of somitogenesis. Embryos transgenic for H2B-GFP were mounted for light-sheet imaging as previously described (Hirsinger and Steventon, 2017). Image stacks were taken every 2.5 min for 8 h. At every fifth time-point, the image stacks are downscaled and an image-based registration was performed with the image stack from five stacks before, with the *x*, *y*, *z* translation values stored. The values are then fed back to the microscope stage to update its position (Fig. 7A). The resulting datasets enable long-term imaging of the tailbud, with continuous displacement of the imaging volume in all directions (Movie 7, right-hand panel). Subsequent 3D registration of these imaging volumes removes the sudden displacements and is suitable for downstream analysis (Movie 7, left-hand panel). The entire dataset can then be observed as either maximally projected image volumes (Fig. 7B; Movie 8) or as slices through the dataset to observe single-cell level resolution (Fig. 7C; Movie 9). A boxed region of shaded background signal reflects the progressive displacement of the microscope stage during imaging (Movies 8 and 9).

At late stages of somitogenesis, the tailbud has segregated into distinct territories that can be defined based on both morphology or gene expression. HCR was performed for *sox2* and *tbxta* (Fig. 7D; yellow and cyan, respectively) and *tbxta* and *tbx16* (Fig. 7E; cyan and red, respectively) at the 28-somite stage. This can be compared with a section through a light-sheet dataset at the same timepoint to reveal how paraxial mesoderm (Fig. 7F; red) and spinal cord (Fig. 7F; blue) can be reliably determined, with a small region of cells still co-expressing *sox2* and *tbxta*, and therefore not assignable in terms of cell fate.

Three independent datasets were tracked using TGMM, with subsequent manual tracking validation and correction performed using Mamut, as described for gastrula stages (Fig. 4). A summary of fates for each movie is shown in Table 1, with the regions tracked for each dataset marked in Fig. 7G. As expected from the proliferation analysis (Fig. 6), no cells in the *sox2/tbxta*-positive region divided in any of the three movies analysed; thus, there is zero chance of an NMp being a bi-fated progenitor during the delayed allocation of *sox2/tbxta*-positive cells to the body axis. We therefore focused our analysis on asking whether mono-fated NMps are arising from a mixed or a sorted progenitor population within the tailbud by mapping retrospectively defined cell fates back to the 21-somite stage (Fig. 7H,I). The Mamut tracking software displays cells close to the *z*-plane of the image as open circles, with coloured dots representing tracks above or below the *z*-plane shown (Fig. 7H). For clarity, we traced all positions onto a diagrammatic outline of the tailbud that shows little cell-mixing of neural (in blue) and mesodermal (in red) mono-fated progenitors (Fig. 7I). Those that were unassignable in terms of fate are coloured yellow. Although some degree of mixing can be seen above the notochord, these mesoderm fated cells are sitting laterally to the spinal cord, as can be seen by the open blue circles and dotted red tracks in this region (Fig. 7H). Together, this shows that although a large Sox2^+^Tbxta^+^ population is maintained in the zebrafish until at least the 21-somite stage (Fig. 5F-H; Movie 6), it is composed of entirely mono-fated progenitor cells that are largely spatially segregated.

**Table 1.**
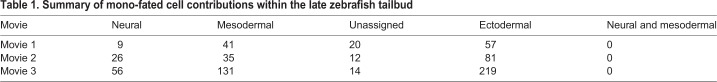
**Summary of mono-fated cell contributions within the late zebrafish tailbud**

## DISCUSSION

**Fig. 8.**
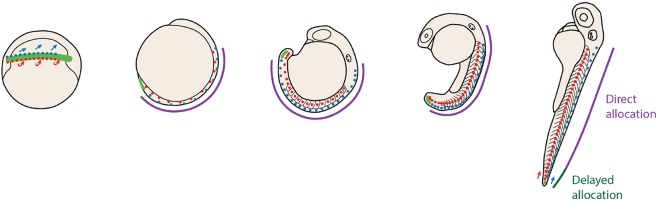
**Zebrafish allocated spinal cord and mesoderm progenitors through a combination of direct and delayed allocation modes.** Diagrams depict zebrafish embryos at progressive stages of development with neuromesodermal competent regions shown in green. Cells either become restricted to spinal cord or paraxial mesoderm fates at early gastrulation (direct allocation; purple) or remain within the tailbud until late somitogenesis (delayed allocation; dark green).

Taken together, these results show that a neuromesodermal progenitor pool is a source for at least a subset of spinal cord cells at all regions of the body axis. Crucially, however, this is not achieved by a bipotent stem cell population but rather by the rapid segregation of neural and mesodermal cell fates during gastrulation. Subsequently, a second population of neuromesodermal progenitors arises along the dorsal wall of the tailbud, co-expressing both *sox2* and *tbxta* (Martin and Kimelman, 2012; this study), and is competent to switch to either neural or mesoderm states in response to changes in Wnt signalling activity (Martin and Kimelman, 2012). However, these are not continually added to the elongating body axis, as previously supposed, but are rather a largely quiescent population of cells that contributes to the last region of the body axis. The low proliferation levels of these cells during somitogenesis are in line with a previous study (Bouldin et al., 2014). Although it is possible that a proportion of cells are bipotent within this population, the low rates of division make it unlikely that their derivatives will have sufficient time prior to the completion of somitogenesis to generate a stem-cell mode of growth as has been observed in mouse embryos (Tzouanacou et al., 2009). Indeed, single cell tracking and retrospective cell fate assignment reveals that the tailbud NMp population consists of a population of fate-restricted and spatially segregated cells. Therefore, we surmize a composite model for spinal cord generation in zebrafish, with spatially and temporally separate pools. The first is directly allocated during gastrulation and is a population of NMps mixed in with a large proportion on monopotent progenitors. The second matches with a previously described tailbud NMp population (Martin and Kimelman, 2012) and has a delayed allocation to only the final region of the larval tail (Fig. 8).

Why is it that zebrafish embryos maintain a tailbud NMp population whose bipotentiality is never realized during normal embryonic development? One possibility is that developmental constraint is acting to conserve a bipotential NMp population across the amniote-anamniote transition, despite large variations in the cellular behaviours that are driving body axis elongation (Steventon et al., 2016; Steventon and Martinez Arias., 2017). Developmental constraint in this case could be acting to maintain the regulatory interactions between transcription factors such as *sox2* and *tbxta*, which maintain a bipotential state, thus leading to a conserved cellular trajectory for NMps (i.e. the series of gene expression states associated with a specific cell fate decision). In contrast, alterations in the multicellular dynamics driving the elongation process may lead to vast alterations in the way this is realized at the cellular level, leading to a divergence in cell lineage (i.e. the series of mother-daughter relationships associated with a specific cell fate decision). Further comparative analyses of NMp populations between chordate model organisms is likely to reveal some important insights into the degree to which cell trajectory and lineage can be separable during evolution and development.

Despite a strong developmental constraint acting upon gastrulation in vertebrates (Abzhanov, 2013), there exists a large degree of morphological variation acting at these stages of development (Duboule, 1994). This variation is largely a consequence of the different strategies of maternal-embryo energetic trade-off that have been adopted during chordate evolution. In the context of mouse, viviparity has led to an internal mode of development within which embryos increase in volume to a large degree, concomitant with establishing their body plan (Steventon et al., 2016). This is in contrast to macrolecithal embryos such as fish, whose energy supplies are contained within an external yolk sac. Such transitions have a great impact on the morphology of gastrulae, which must adopt their shape according to the physical constraints of yolk size and extraembryonic structures. Furthermore, external modes of development provide a selective advantage for developmental strategies that favour a rapid development to swimming larval stages in order that they escape predators and find food. Zebrafish undergo a highly rapid mode of development, and develop their full complement of somites, prior to the development of a vascular system that is efficient at accessing nutritional supplies and allowing them to increase in mass. We propose that by shifting the allocation of spinal cord and mesodermal progenitors to early gastrulation stages, this has facilitated a rapid convergence and extension-based mode of axial elongation. Furthermore, the pool of NMps within the tailbud demonstrates a conservation of a tailbud progenitor pool that could allow for increased flexibility according to organism level heterochronies in the rates of growth. This hints at a novel evolutionary developmental mechanism that we term ‘growth-mode adaptability’. This proposes that specific cellular trajectories (such as the emergence of spinal cord from a neuromesodermal cell state) are conserved in evolution, yet highly adaptable in terms of their timing of cellular decision making and modes of growth.

## MATERIALS AND METHODS

### Animal lines and husbandry

This research was regulated under the Animals (Scientific Procedures) Act 1986 Amendment Regulations 2012 following ethical review by the University of Cambridge Animal Welfare and Ethical Review Body (AWERB).

### Scartrace

Scarred zebrafish (injected with RNA Cas9 or protein Cas9 and gRNA against GFP) were made as described by Junker et al. (2017preprint) and Alemany et al. (2018). Scarred zebrafish (around 1 year old) were anaesthetized, sacrificed and small parts of body structures were dissected and collected individually [a full list of all dissected body structures and barcodes (BC) are available in Tables S1-S6]. Genomic DNA was isolated from the body structures using DNeasy Blood and Tissue Kits (Qiagen, 69506) and scars were amplified and sequenced using the ScarTrace bulk protocol as described by Junker et al. (2017preprint). Scars were mapped with the Burrows–Wheeler Aligner as described by Alemany et al. (2018). Raw sequencing data (as fastq files) and mapped scars tables have been deposited in GEO under accession number GSE121114. After mapping, to filter out potential sequencing errors, the scar percentage was computed in normalized histogram with 100 bins. Scars were only kept when their fraction was at least 10× higher than the minimum detected scar fraction. To filter out fish with inefficient scarring, we determined the scarring efficiency as the number of scars after filtering and the mean unscarred GFP percentage across all organs per fish. Only fish with less than 50% unscarred GFP and more than 100 scars were kept (these are fish R1-R3 and fish P1-P3). Afterwards, to ensure we kept scars that were made once in the developing embryo, we filtered out all scars that we detected in other fish and in the dynamics experiments from Alemany et al. (2018). This filtering step ensured that we kept only rare scars, with a probability of *P*=10^–5^ of being made. After filtering, we summed scars from technical replicas of the same dissected body structure. As a last processing step, we binarized the filtered scars (Tables S7-S12) and computed the distances between body structures using a weighted distance measure for sparse samples (information weighted sparse sample distance, IWSS). IWSS incorporates both the matches and mismatches in an information-weighted manner to incorporate all available information in sparsely sampled vectors. The IWSS distances between the organs are shown as log distances in heatmaps (Fig. 1B and Fig. S1) and bootstrapped trees (Fig. 1C and Fig. S1). Heatmaps were plotted using the python seaborn package with the settings method=‘average’ and metric=‘euclidean’. Trees were bootstrapped 100 times using the R function boot.phylo and prop.clades from the ‘ape’ package.

### Photolabelling and time-lapse microscopy fate mapping

Zebrafish wild-type embryos were injected at the one-cell stage with 200 pg of nuclear-targeted Kikume (a kind gift from Ben Martin, Stony Brook University, NY, USA). Photolabelling was performed as described previously (Steventon et al., 2016).

For fate-mapping experiments at gastrulation stages, six to ten 30% or 50% epiboly stage embryos were embedded in a drop of low gelling point agarose (1% w/v in E3 media) at the bottom of a MatTek petri dish (1.5 glass coverslip bottom) and imaged using a 10× air objective (NA=0.45). Time lapse imaging of labelled embryos was performed using a Zeiss LSM 700 confocal microscope equipped with a temperature-controlled chamber set at 28°C. In our setup, nearly complete photoconversion of NLS-KikGR was obtained by scanning the 405 nm laser at 10-15% laser power on a circular user-defined region of the embryo. Photolabelling efficiency was directly visualized by simultaneously capturing both NLS-KikGR emissions on two different PMTs. After labelling of all embryos, overnight multidimensional acquisition of low resolution stacks for each embryo was set up with an interval of 10 min. The experiment was stopped the morning after embryos had reached the 16-somite stage, at which they were dissected out of the agarose, individually dechorionated, re-embedded and imaged at higher *z*-resolution. The excitation power of 488 and 561 nm lasers was kept below 12% throughout time-lapse acquisition and further lowered after dechorionation.

To quantify label contribution to neural or paraxial mesoderm tissues, as well as their axial spreading, high-resolution datasets of 16-somite stage embryos were segmented and cells counted using the surface and spot functions in Imaris v8.3 (bitplane.com).

For postgastrulation labelling, embryos were mounted in 2% methycellulose in E3. Mounting in low gelling point agarose in E3, as described by Hirsinger and Steventon (2017), was performed for live imaging of post-gastrulation embryos.

### Scanning light-sheet imaging microscope with online stage adjustment

The additional light-sheet imaging was performed using a home-build scanning light-sheet microscope in an upright geometry, where the excitation and emission lenses were each tilted 45° to the vertical axis to allow for horizontal sample placement on a *xyz*-translation stage. The sheet was generated using 2-4% laser output power at 488 nm (200 mW maximum output power), delivered to the sample using a Nikon 10×, 0.3NA water-immersion objective and a galvo scanning system (Cambridge Technology). The sheet thickness determined before the experiment in dye solution was ∼2 μm at the waist. Fluorescence was collected with a Nikon 40×, 0.8 NA water-immersion objective, using a 525/50 nm (Chroma Technologies) emission filter in front of the sCMOS camera (Hamamatsu Flash 4). The position of the detection lens was synchronized with the position of the excitation sheet using a long travel piezo objective positioner (Physik Instrumente).

To allow automated long-term imaging of the structurally changing sample, an automated feature of the tracking method was implemented in the bespoke control software written in Labview. Data handling was performed by calling MATLAB scripts from within the software. The goal was to keep the area of interest in the field of view so that further registration could happen offline. During time-lapse acquisition, all image stacks are downsampled online to 1×1×1 μm^3^ voxel size to allow for efficient data handling. For every 5th timepoint, a three-dimensional phase correlation is calculated between the current downsampled stack and a reference stack. From this, the necessary *xyz*-stage shift is determined and sample moved accordingly. The next image stack replaces the previous reference stack to take into account overall changes. All necessary computation steps were optimized so that they could be completed in between timepoints in order not to delay image acquisition. After the completed time-lapse image acquisition, consecutive image stacks were registered using custom MATLAB scripts employing the same 3d-phase-correlation algorithm.

### Hybridization chain reaction and immunohistochemistry

Five antisense DNA probes were designed against the full-length zebrafish *sox2* and *ntla* mRNA sequence as described by Choi et al. (2014). Embryos incubated in either embryo medium were fixed 4% PFA at 4°C and then stained according to Choi et al. (2014). HCR was combined with immunohistochemistry for phospho-histone H3 for identification of mitotic cells at the point of fixation. Embryos underwent HCR followed by blocking in 2% Roche blocking reagent, 5% donkey serum in maleic acid buffer. A mouse anti-pH3 antibody (abcam, ab14955) was incubated overnight at 4° followed by a secondary anti-mouse conjugated to an Alexa 488 fluorophore (ThermoFisher Scientific, A32723). Imaging was performed on a Zeiss LSM700 confocal with identical imaging parameters: *z*-step 0.5683 μm; 1024×1024 resolution; 63× oil objective NA 1.4; 35% laser power at 488 nm; Gain 661; Digital offset −2; Pixel dwell time 3.12 μs.

### MATLAB script for trajectory selection

The custom-written MATLAB script imports trajectories saved in TGMM software format (Amat et al., 2014) and visualizes the position of the detected cells at two user-defined points in time. Through GUI the user selects the cells of interest and subsequently the trajectories of the respective cells are plotted in red. The trajectories retained also include those of the daughter cells originating from the originally selected cells at any timepoint. An (optional) post-processing step eliminates the cells on the opposite side of the embryo from the user's viewpoint, in case they were inadvertently selected. The selected trajectories can be saved in the TGMM software format or as a MATLAB ‘.mat’ file.

### *In silico* fate mapping

The light-sheet dataset used for the lineage analysis, as well as the TGMM automated tracking (Amat et al., 2014), has been described by Shah et al. (2017preprint). Trajectories selected at timepoint 30 (corresponding to 30% epiboly) were exported as TGMM output .xml file using our MATLAB script. Position of cells analogous to those analysed in fate-mapping experiments were obtained by comparing the 30% epiboly stage with the 75% epiboly stage, at which the embryonic shield is visible. TGMM data was imported into MaMuT (Wolff et al., 2018) for visualization and validation of tracks. Lineages were followed using the Track Scheme module and inspected by overlaying the tracking data on top of the original images in Mamut viewer. Germ layer segregation of cells along tracks was determined based on the expression of the *mezzo:eGFP* mesodermal reporter. Endodermal cells, expressing *sox17::YFP*, were excluded. Spinal cord contribution of non-mesodermal cells was manually verified using Mamut.

## Supplementary Material

Supplementary information
