# Correction: Neuromesodermal progenitors are a conserved source of spinal cord with divergent growth dynamics (doi: 10.1242/dev.166728)

**DOI:** 10.1242/dev.175620

**Published:** 2019-01-22

**Authors:** Andrea Attardi, Timothy Fulton, Maria Florescu, Gopi Shah, Leila Muresan, Martin O. Lenz, Courtney Lancaster, Jan Huisken, Alexander van Oudenaarden, Benjamin Steventon

Light-sheet imaging data associated with *Development* (2018) **145**, dev166728 (doi: 10.1242/dev.166728) are now available in the Image Data Repository with an explanatory Data Note hosted by Wellcome Open Research.

The corrected Data availability section is shown below and both the online full-text and PDF versions have been updated.

