# Correction: Neuromesodermal progenitors are a conserved source of spinal cord with divergent growth dynamics

**DOI:** 10.1242/dev.195610

**Published:** 2020-09-08

**Authors:** Andrea Attardi, Timothy Fulton, Maria Florescu, Gopi Shah, Leila Muresan, Martin O. Lenz, Courtney Lancaster, Jan Huisken, Alexander van Oudenaarden, Benjamin Steventon

There was an error in Development (2018) 145, dev166728 (doi:10.1242/dev.166728).

In Fig. 6, the graph in panel G was duplicated in panel I.

**Corrected:**

**Fig. 6.**
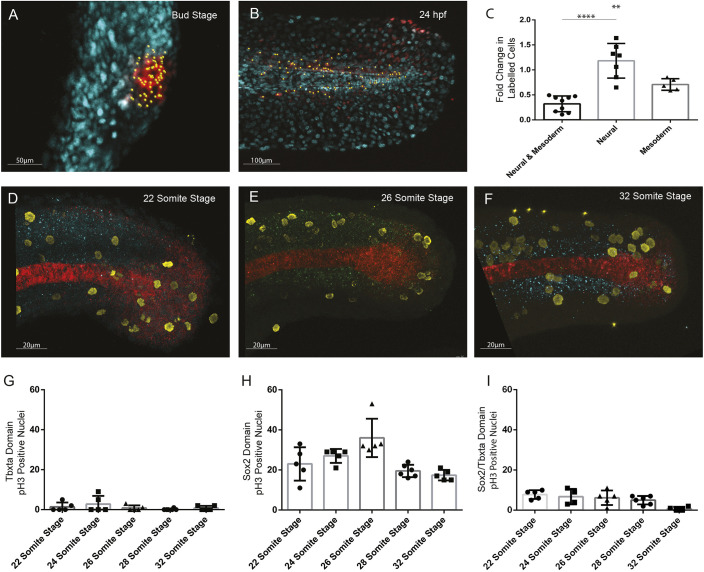
**Quantification of cell division in tailbud NMps.** Quantification of increase in number of cells photolabelled using nls-kikume from (A) bud stage through to (B) 24 hpf. (C) Fold-change increase in labelled clone number changes depending on the labelled progenitor type, with regions contributing only to neural tissue undergoing most clonal expansion and bipotent progenitors undergoing the least clonal expansion. (D-F) Replicating cells stained using phospho-histone H3 (pH3) as a marker of mitotic cells (yellow) with bipotent NMps identified through co-expression of Sox2 (blue) and Ntl (red) at the (D) 22-somite stage, (E) 26-somite stage and (F) 32-somite stage. (G-I) The frequency of Ph3-positive nuclei in the (G) Ntl-positive expression domain, including notochord, (H) Sox2-positive expression domain, and (I) Ntl and Sox2-positive NMp domain. Two-tailed Student's *t*-test. **P<0.01, ****P<0.0001.

**Original:**

**Fig. 6.**
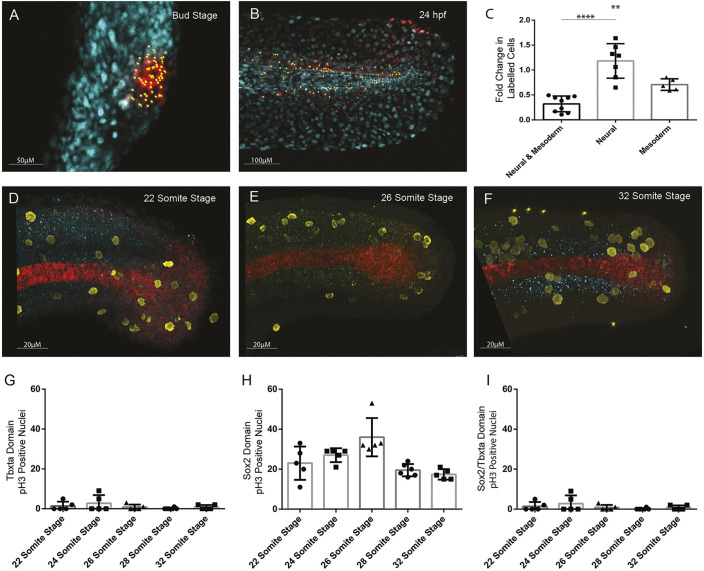
**Quantification of cell division in tailbud NMps.** Quantification of increase in number of cells photolabelled using nls-kikume from (A) bud stage through to (B) 24 hpf. (C) Fold-change increase in labelled clone number changes depending on the labelled progenitor type, with regions contributing only to neural tissue undergoing most clonal expansion and bipotent progenitors undergoing the least clonal expansion. (D-F) Replicating cells stained using phospho-histone H3 (pH3) as a marker of mitotic cells (yellow) with bipotent NMps identified through co-expression of Sox2 (blue) and Ntl (red) at the (D) 22-somite stage, (E) 26-somite stage and (F) 32-somite stage. (G-I) The frequency of Ph3-positive nuclei in the (G) Ntl-positive expression domain, including notochord, (H) Sox2-positive expression domain, and (I) Ntl and Sox2-positive NMp domain. Two-tailed Student's *t*-test. **P<0.01, ****P<0.0001.

Both the online full-text and PDF versions have been updated.

The authors apologise to readers for this error, which does not alter the conclusions of the manuscript.

