# Supplementary material for: Neuromesodermal progenitors are a conserved source of spinal cord with divergent growth dynamics
Source: Development. 2018 Nov 9;145(21):dev166728. doi: 10.1242/dev.166728 (PMC6240315; doi:10.1242/dev.166728)
Supplement: Supplementary information [file develop-145-166728-s1.pdf]

Supplementary Information

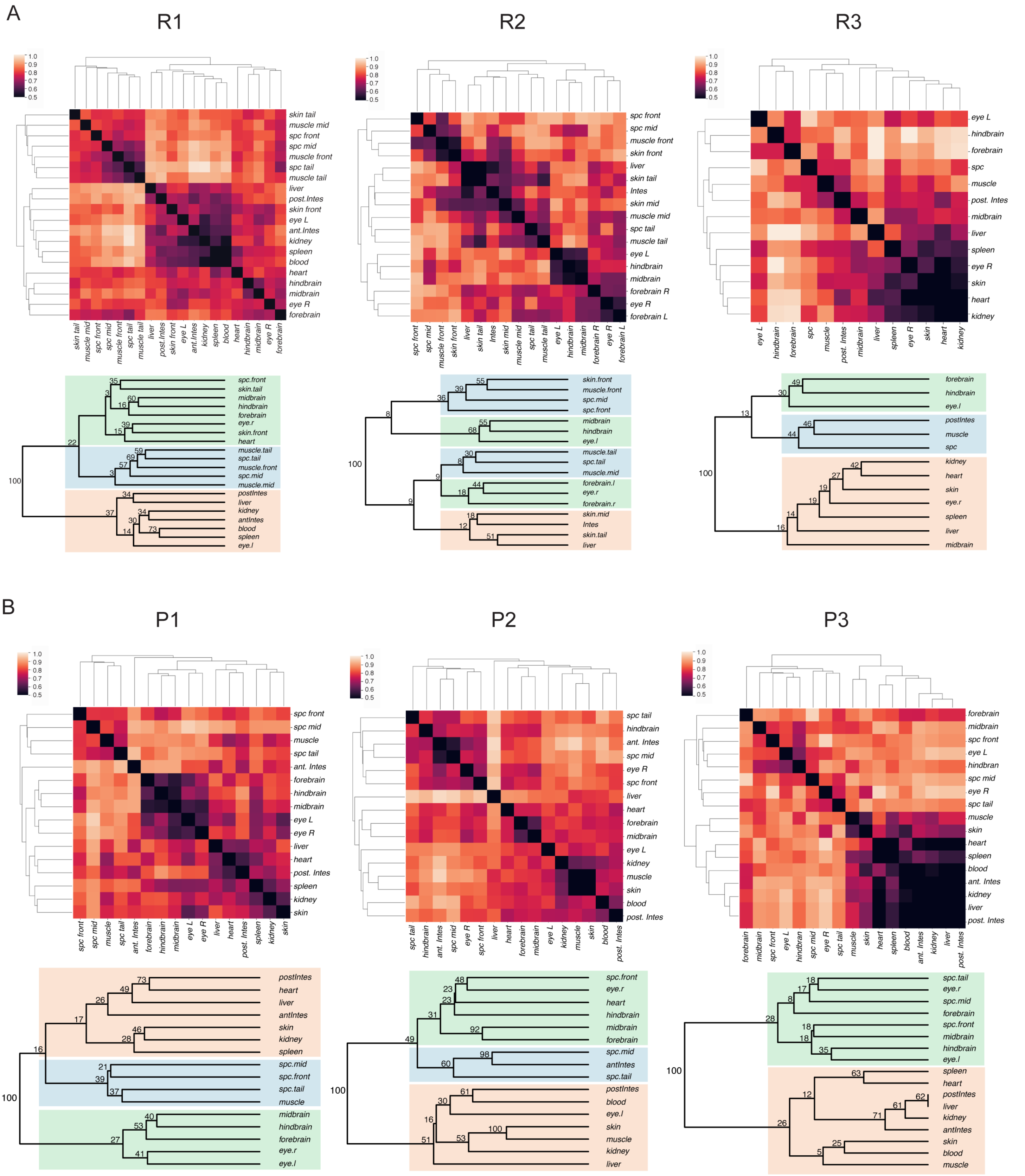

Development • Supplementary information

Figure S1. ScarTrace Clustering and trees of weighted distances between body structures of **(A)** Cas9 RNA injected fish R1 (as shown in Figure 1), R2 and R3 and **(B)** Cas9 protein injected fish P1, P2 and P3. injected fish and P1 to P3. Each tree was bootstrapped 100 times and the final proportion of clades (clade support values) are shown at each node of a clade. Color in the background are a guide to the eye for following the close association of spinal cord and mesodermal body structures across the anterior to posterior axis, that is distinct from brain regions in R2, R3 and P1. antIntes = anterior intestine, postIntes = posterior intestine, l. = left, r. = right, Spc. = Spinal cord

Development • Supplementary information

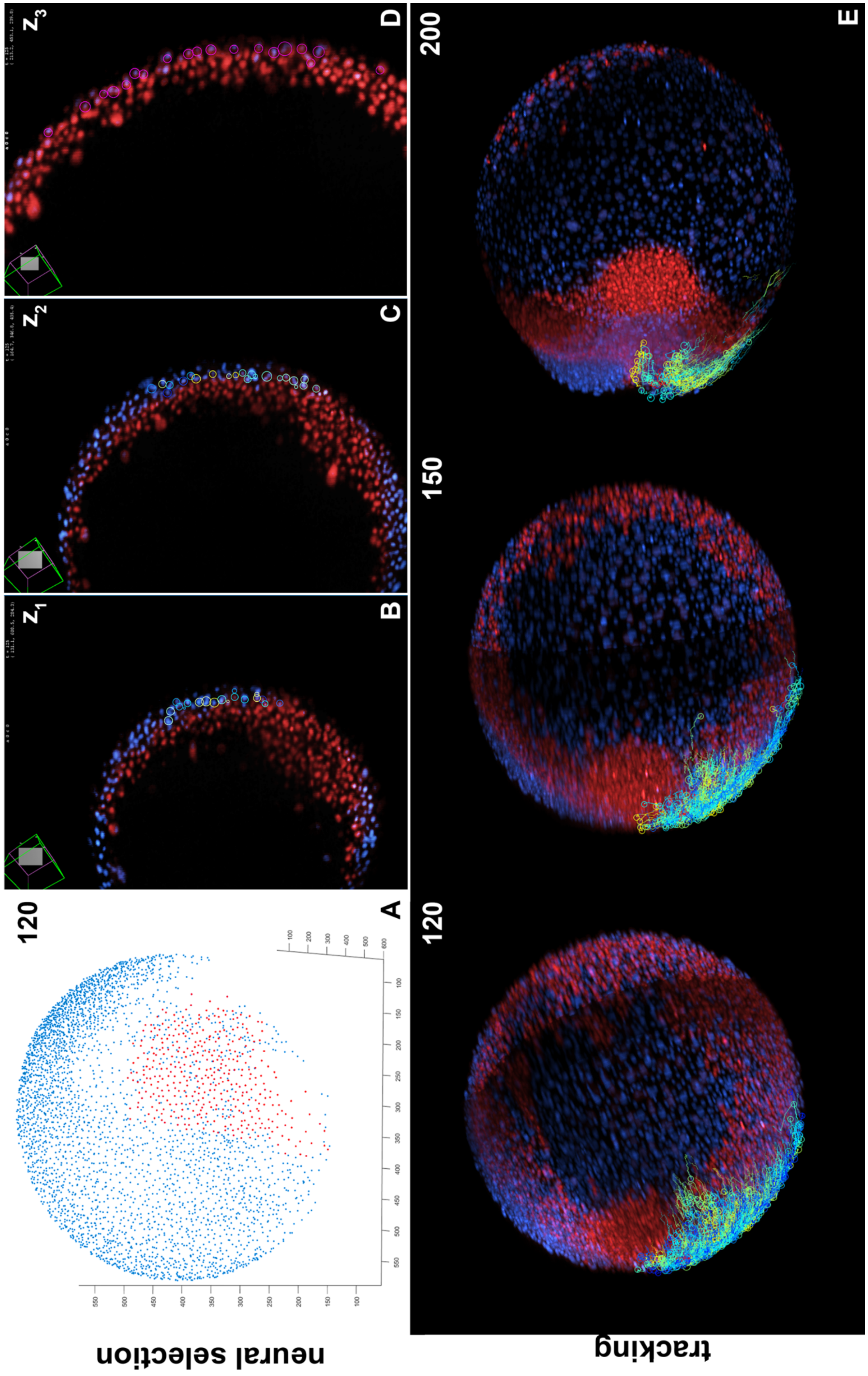

Development • Supplementary information

Figure S2. Spinal cord allocation of upper marginal cells. (A) Cells of the upper marginal label shown in Figure 3 are selected (red spots) and followed from timepoint 120. (B-D) Mamut Viewer visualisation of the selection at three different optical sections shows that cells lack *mezzo* expression and are still localised in the outer layer of the blastoderm. (E) Further tracking of these ectodermal cells shows their migration towards the embryonic anteroposterior axis and final arrangement in one of the two arcs of cells that converge to form the spinal cord. Images in E depict partial maximum intensity projections to exclude the head region at the indicated timepoints.

Supplementary Tables 1-6. All dissected body structures and barcodes. For each Cas9 RNA injected fish (R1-R3) and Cas9 protein injected fish (P1-P3) we show a separate table listing the barcodes ("BC" column) needed to match reads from a sequenced library (one library per fish, can be found under GEOXXXXXX) to the corresponding dissected body structure ("Organ" column).

Click here to Download Tables S1 - S6

Supplementary Tables 7-12. Binarized scars tables. For each scarred fish we are providing the binarized scars tables we are obtaining after mapping, filtering and binarizing as described in Materials and Methods. These tables were used as input to calculate the IWSS distances between the dissected body structures shown in form of heatmaps and trees in Fig. 1 and Fig S1. The rows of the tables show each scar as a CIGAR string and the columns the disstected body structure.

Click here to Download Tables S7 - S12

Development • Supplementary information

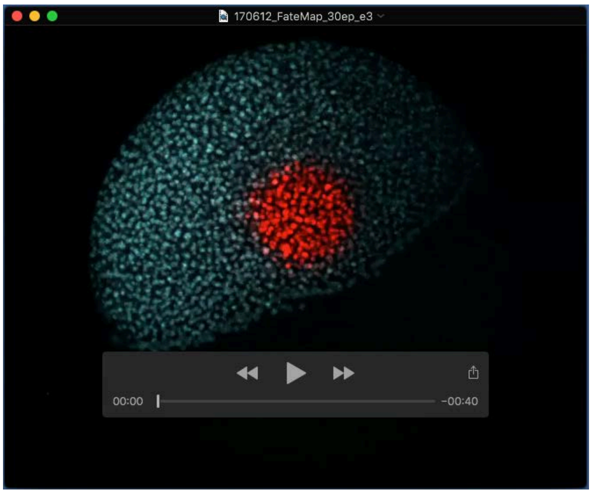

Movie 1. Photolabel at 50% epiboly with large contributions to both spinal cord and paraxial mesoderm

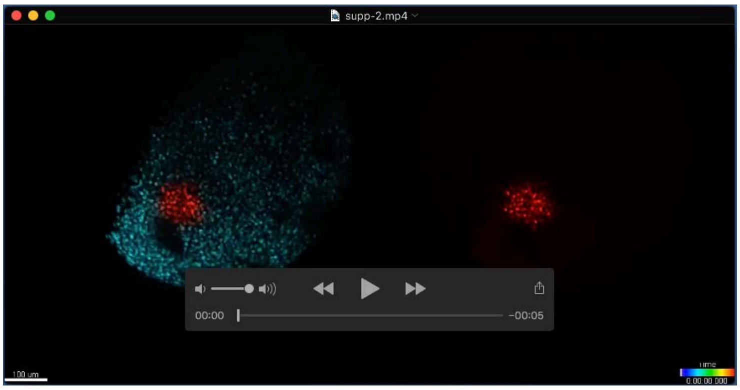

Movie 2. Convergence and extension of spinal cord fated photo-labeled cells at 50% epiboly

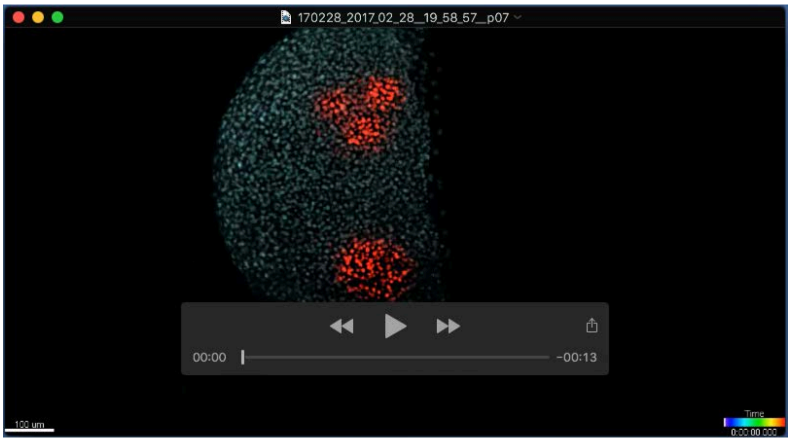

Movie 3. Photolabels across the marginal zone contribute to large portions of the anterior-posterior axis

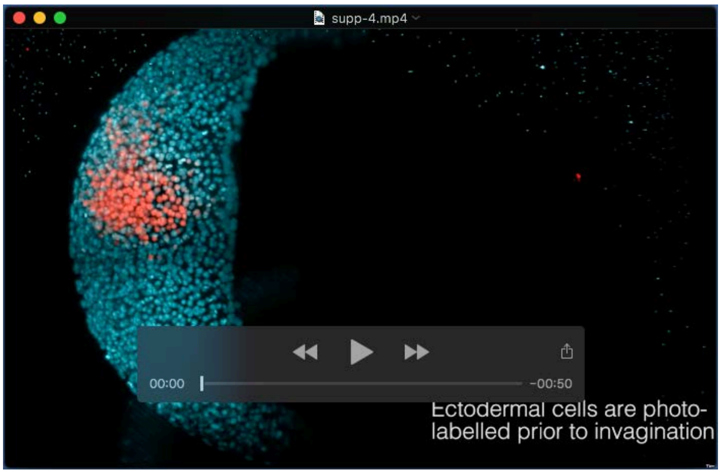

Movie 4. Light-sheet movie of a 50% neuromesodermal fated photolabel.

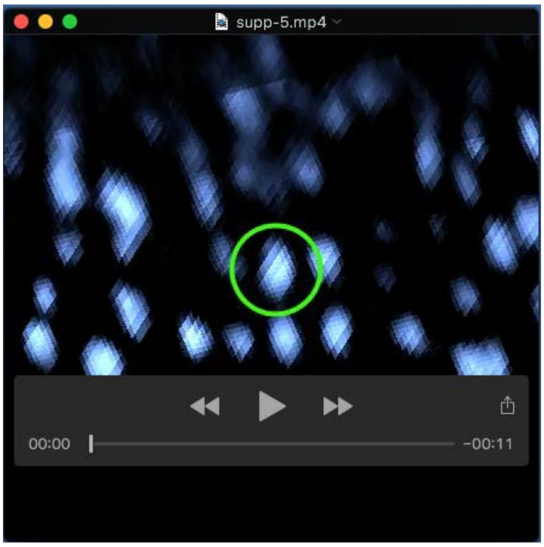

Movie 5. Manual verification of a mono-fated mesodermal progenitor.

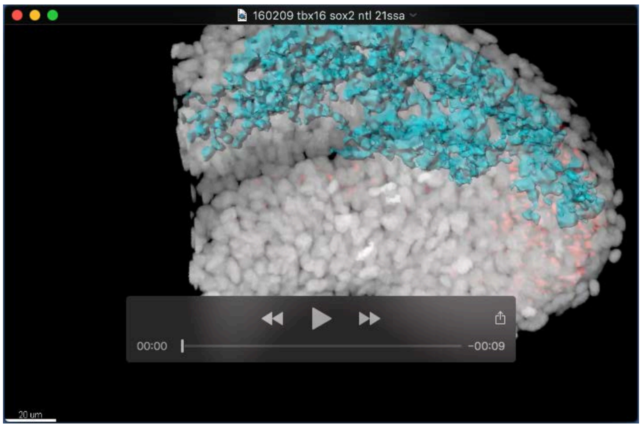

Movie 6. 3D segmentation of Sox2 and Tbxta positive cells within the zebrafish tailbud

Development • Supplementary information

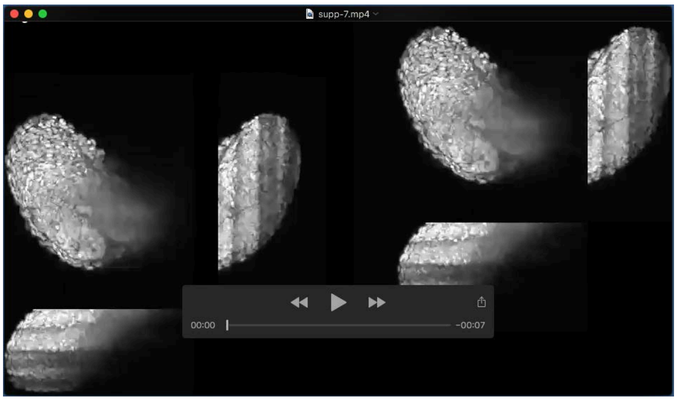

Movie 7. Sections through a tailbud-stage light-sheet movie before and after image registration

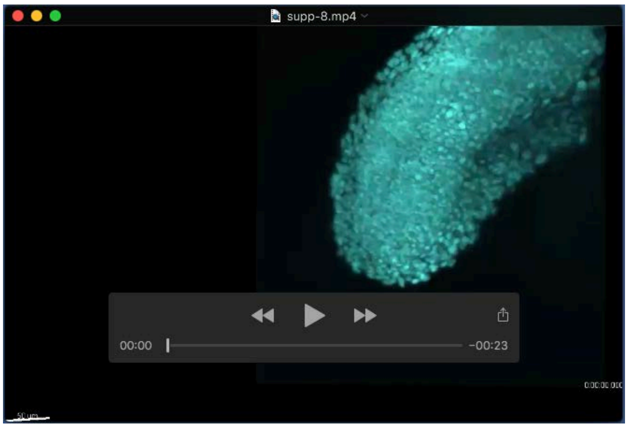

Movie 8. Maximum projection of a registered tailbud movie

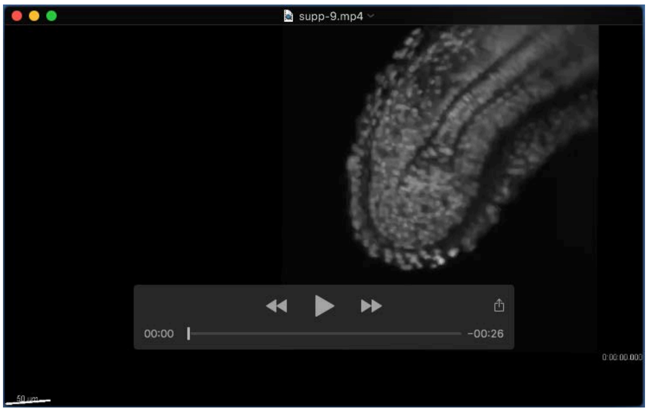

Movie 9. Midline section of a registered tailbud movie

Development • Supplementary information
